# Optical mapping of neuronal activity during seizures in zebrafish

**DOI:** 10.1038/s41598-017-03087-z

**Published:** 2017-06-08

**Authors:** L. Turrini, C. Fornetto, G. Marchetto, M. C. Müllenbroich, N. Tiso, A. Vettori, F. Resta, A. Masi, G. Mannaioni, F. S. Pavone, F. Vanzi

**Affiliations:** 1 European Laboratory for Non-Linear Spectroscopy (LENS), Sesto Fiorentino, Italy; 2 Department of Biology, University of Florence, Sesto Fiorentino, Italy; 3 National Institute of Optics (INO), National Research Council (CNR), Sesto Fiorentino, Italy; 4 Department of Biology, University of Padova, Padova, Italy; 5 Department Neurofarba, University of Florence, Florence, Italy; 6 Department of Physics, University of Florence, Sesto Fiorentino, Italy

## Abstract

Mapping neuronal activity during the onset and propagation of epileptic seizures can provide a better understanding of the mechanisms underlying this pathology and improve our approaches to the development of new drugs. Recently, zebrafish has become an important model for studying epilepsy both in basic research and in drug discovery. Here, we employed a transgenic line with pan-neuronal expression of the genetically-encoded calcium indicator GCaMP6s to measure neuronal activity in zebrafish larvae during seizures induced by pentylenetretrazole (PTZ). With this approach, we mapped neuronal activity in different areas of the larval brain, demonstrating the high sensitivity of this method to different levels of alteration, as induced by increasing PTZ concentrations, and the rescuing effect of an anti-epileptic drug. We also present simultaneous measurements of brain and locomotor activity, as well as a high-throughput assay, demonstrating that GCaMP measurements can complement behavioural assays for the detection of subclinical epileptic seizures, thus enabling future investigations on human hypomorphic mutations and more effective drug screening methods. Notably, the methodology described here can be easily applied to the study of many human neuropathologies modelled in zebrafish, allowing a simple and yet detailed investigation of brain activity alterations associated with the pathological phenotype.

## Introduction

Epilepsy is one of the most common neurological disorders worldwide and is characterized by recurrent spontaneous bursts of neuronal activity, known as seizures. Genetic mutations, such as various ion channelopathies^1^, alteration of neuronal migration, or protein degradation defects in neurons (e.g. Angelman syndrome) can be frequently associated with epilepsy^2, 3^. Moreover, epileptic seizures can occur as a consequence of a variety of insults, such as brain tumours, injury, stroke, or neurodegeneration. Despite a wide range of anti-epileptic drugs (AEDs) currently available for treatment of epilepsy^4^, approximately 35% of patients fail to respond adequately to therapy^5, 6^. In the last years, most of the research on AEDs has been performed using rodent models. These models have significantly contributed to increase our comprehension of epilepsy but suffer from limitations for the identification of new AEDs, regarding, for example, the throughput of drug screening and the time required to generate new transgenic lines to model specific epileptic pathogenic mechanisms. Therefore, to further increase our understanding of basic mechanisms underlying epilepsy and to develop novel treatments new experimental approaches and new animal models^7, 8^ are needed. Over the past decade, zebrafish (*Danio rerio*) has rapidly emerged as an important model organism in genetics and in biomedical research, with strong applications in translational neurosciences^9^. There are considerable advantages in utilizing zebrafish for the study of neurobiology, neuropathology and for the development of new therapeutic approaches. Zebrafish, in fact, exhibits external embryonic development (making it easy to manipulate), high fecundity rate (providing large and homogeneous samples that are ideal for high-throughput screenings), and a fully sequenced genome, with about 70% of human genes having at least one orthologue in zebrafish^10^, making it suitable for the implementation of models of human pathologies, including epilepsy. Moreover, the brain of this teleost is characterized by regions structurally similar to those in the mammalian brain and with similar neurochemistry^11, 12^. Finally, the optical transparency of larvae during the first week of development enables *in vivo* imaging and is undoubtedly one of the strongest points of this animal model both in basic research and in the field of neurological disease analysis.

Several recent studies revealed that pentylenetetrazole (PTZ) can be successfully used to induce seizure-like behaviour in zebrafish larvae, making this pharmacological approach suitable to study several kinds of human epilepsy in this animal model^13, 14^. Also, many genes involved in epilepsy have been investigated in zebrafish models, including mutants showing resistance to seizures^15, 16^, hypersensitivity to convulsant drugs or even spontaneous seizures^17^. Thus far, epileptic seizures have been detected and characterized in zebrafish either as behavioural alterations or by means of direct electrographic recordings. Behavioural measurements rely on the recording of swimming activity by time-lapse videos and tracking of the larva position in time^13, 18, 19^. During seizures, larvae exhibit a variety of behaviours classified in different epileptic stages^13^. This type of measurements has been used to characterize chemically-induced convulsions and to screen potential anti-convulsant drugs, capable of reversing the effects of PTZ^20^.

However, a detailed study of the mechanisms of epilepsy requires measuring the neuronal activity underlying seizures. The most used method to this end is the electrographic recording of activity in large areas of the larval brain via extracellular field recordings. These studies have proved effective in detecting brain electrical activity and its alterations, which correlate with seizures^13, 18, 21^. However, a full comprehension of the genesis and propagation of seizures would ultimately require measuring activity at cellular resolution. The measurement of activity with high spatial resolution would most likely also improve the efficacy of drug screenings for specific compounds by discarding those molecules acting on undesired target brain areas.

An important advancement in the measurement of neuronal activity has been represented by the development of optical methods based on fluorescent calcium indicators, taking advantage of the correlation between intracellular calcium concentration and frequency of spiking. Fast and sensitive genetically-encoded calcium indicators (such as GCaMP^22, 23^) represent a powerful tool for the investigation of neuronal dynamics with up to single action potential sensitivity^22^. Moreover, genetically targeting the expression of the indicator allows selecting the population of cells probed (for example neurons versus glial cells, or subpopulations of neurons within the central nervous system).

Compared to electrophysiological recordings, in which an electrode is typically introduced into the tissue, the use of light has several advantages. Most prominently, optical measurements are much less invasive and allow the parallel measurement of activity on large populations of neurons (up to the whole brain) with a resolution that can probe single cells. Indeed, in recent years, the use of GCaMP in zebrafish has been proven highly valuable for the measurement of neuronal activity across the whole brain of the larva with the capability of identifying about 80% of its 100,000 neurons^24, 25^.

Here, we present optical recordings of whole brain activity in zebrafish larvae expressing the genetically-encoded calcium indicator GCaMP6s under the pan-neuronal promoter elavl3^26^. These measurements are employed to quantify and map brain activity during the treatment with the well-characterized convulsant agent PTZ. The method proves highly effective in detecting seizures of different intensities and patterns as induced by different PTZ concentrations, probing the involvement of different brain areas in different conditions. We also demonstrate a combined measurement of optical neuronal activity measurements and locomotor behaviour of the larva, proving that optical measurements are more sensitive than behavioural assays in detecting increased activity linked with convulsive or subclinical epileptic seizures. Finally, we implement GCaMP6s measurements of brain activity in a high-throughput assay, demonstrating that combined calcium indicator fluorescence and swimming behaviour measurements can be performed on a large number of larvae undergoing different pharmacological treatments. This approach opens the way to screening methods in which the behavioural information is complemented with an actual measurement of neuronal activity, allowing a more complete view of drug candidates’ effectiveness.

## Results

### GCaMP6s optical measurements show basal and PTZ-altered activity in the zebrafish brain

**Figure 1:**
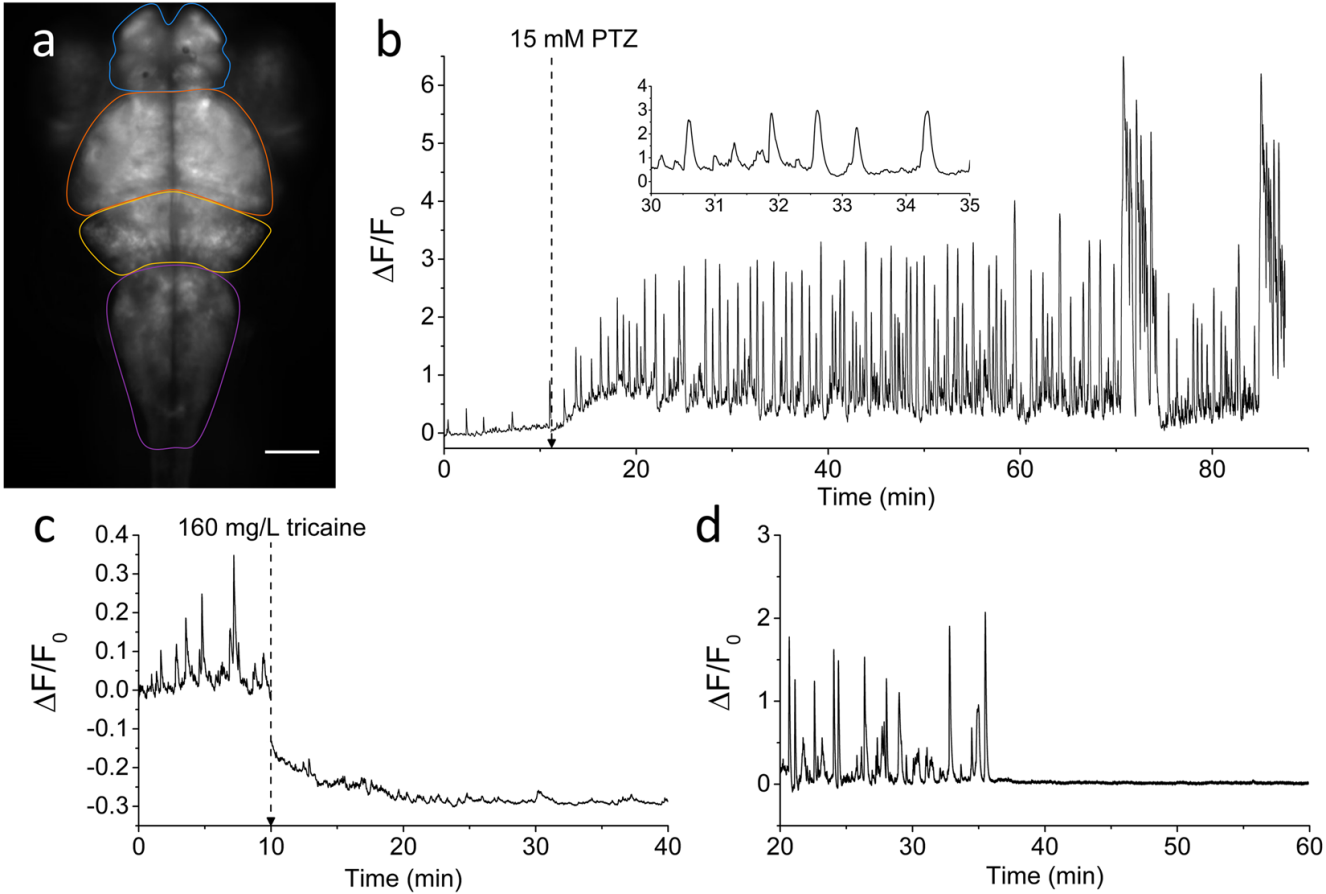
Brain activity recording with GCaMP6s. (**a**) Fluorescence image of the encephalon of a 4 dpf larva expressing GCaMP6s under elavl3 promoter. Scale bar: 100 µm. The four main zebrafish brain regions used for further analysis in the paper are highlighted (Telencephalon, blue; Optic tectum, orange; Cerebellum, yellow; Medulla, purple). (**b**) Time trace of fluorescence (ΔF/F_0_, see Methods for detail) in control conditions and after addition of 15 mM PTZ (at the time indicated). The fluorescence data are integrated over the whole brain of the larva and sampled at 5 Hz. The inset shows a shorter time interval of the trace to better display calcium peak shape and regularity. (**c**) ΔF/F_0_ trace of an experiment in which tricaine (160 mg/L) was added at the time indicated and the subsequent decay of activity was monitored. The ΔF/F_0_ values become negative after tricaine addition since the F_0_ reference level was measured in control conditions (see Methods for details). (**d**) ΔF/F_0_ trace showing sharp brain activity decay in a larva simultaneously treated with 15 mM PTZ and 5 μM TTX. After approximately 35 min upon drugs application, TTX action totally suppressed neuronal seizure activity induced by PTZ.

For this work, a zebrafish transgenic line was generated (as described in Methods) to express GCaMP6s^22^ in all CNS neurons. Pan-neuronal expression in the Tg(elavl3:GCaMP6s) zebrafish encephalon provided a strong signal that maps throughout the whole larval brain, allowing us to clearly distinguish different anatomical areas of the encephalon such as the telencephalon, the optic tectum, the cerebellum and the medulla (Fig. 1a). During the analysis of brain activity, the larvae were mounted under the microscope in a setup allowing solution exchanges (see Methods), so that each larva could be monitored in control conditions for about ten minutes, before adding PTZ at the desired concentration. This experimental protocol enabled the direct measurement of the onset of the compound activity as well as its steady effect at prolonged exposures. Figure 1b shows a typical experiment in which 15 mM PTZ is added after 11 minutes of control recording. The ΔF/F_0_ trace (see Methods) clearly shows an increase of the baseline developing early after PTZ addition. Compared to control (where few small peaks of spontaneous activity are visible, with a maximum amplitude of about 0.4), the effect of PTZ developed gradually over about ten minutes and reached a steady convulsion activity with large and regularly paced calcium spikes. In the trace shown in Fig. 1b, during the 50–60 minutes following the onset of PTZ effect, activity was regularly paced, with ΔF/F_0_ maximum amplitudes of 2–3. With more prolonged exposure to PTZ even larger bursts became evident (ΔF/F_0_ up to 5–6).

The data shown in Fig. 1b demonstrate the high sensitivity of our recording method to the convulsant effects of PTZ. Moreover, these experiments demonstrate that different stages of convulsions can be recognized in the calcium trace, depending on both amplitude and frequency of the fluorescence peaks. It is also interesting to consider the signal baseline and its dynamics: due to projection of the fluorescence signal on one plane and the integration over the whole brain, the measured baseline can indeed be interpreted as a measurement of basal brain activity of the larva. Our measurements are very sensitive to this parameter, as demonstrated by the sharp and rapid increase of the baseline upon exposure to PTZ. To further test this sensitivity, we performed measurements of activity upon treatment with tricaine, an anaesthetic commonly used to immobilize zebrafish larvae. Figure 1c shows the marked decay of basal neuronal activity associated with the tricaine treatment. The use of a specific blocker of voltage-dependent Na^+^ channels (Tetrodotoxin, TTX) also induced complete suppression (after about 35 minutes of treatment) of the calcium signal in our measurements in the presence of 15 mM PTZ (Fig. 1d), demonstrating that the signal is entirely due to neuronal firing activity. Thus, GCaMP integrated measurements are effective in monitoring both basal activity and dynamics due to alterations such as seizures or drug treatments.

### GCaMP6s fluorescence measurements are sensitive to different PTZ concentrations

**Figure 2:**
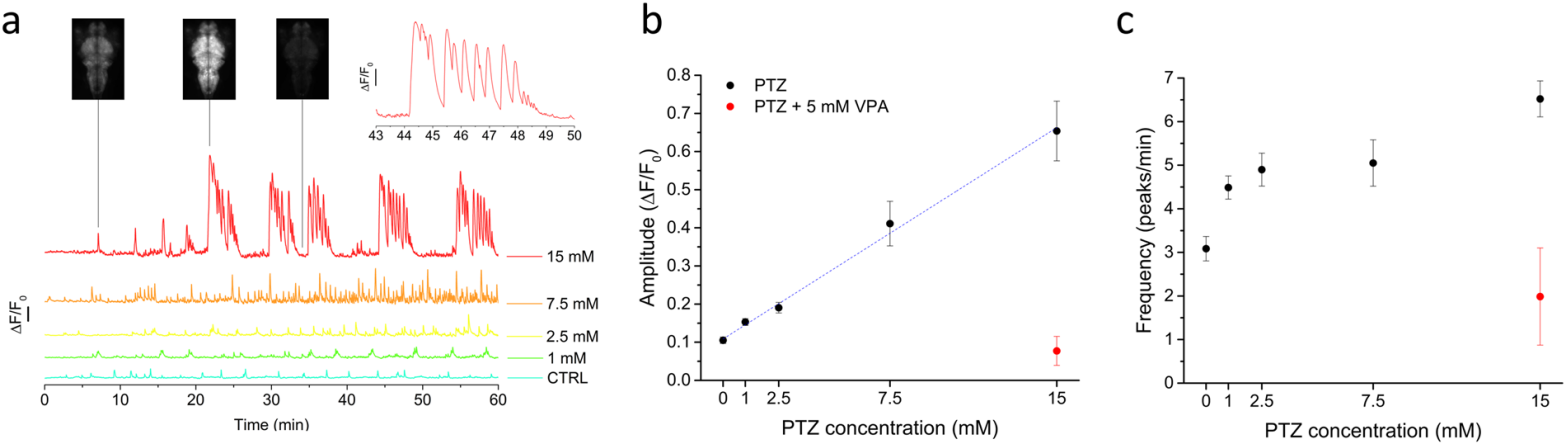
Comparison of the effects of different PTZ concentrations on brain activity. (**a**) ΔF/F_0_ is plotted as a function of time for control conditions and increasing PTZ concentrations as indicated. Upper left panels show images acquired at the points indicated during the recording at 15 mM PTZ, demonstrating the large dynamic range of the signal and lack of saturation even at the highest recorded levels. The inset on the upper right shows a single cluster of closely spaced calcium spikes occurring at 15 mM PTZ and displayed on a magnified time scale. ΔF/F_0_ calibaration bar: 100%. (**b**) Mean amplitude of peaks detected over one hour registration for each larva recorded (see Methods for details about automatic peak detection). The red symbol represents measurements performed on larvae treated with 15 mM PTZ after a pre-incubation of one hour in 5 mM Valproic acid (VPA). The linear regression through the PTZ-only measurements (dashed line) produces a slope of 0.037/mM, Pearsons’s r = 0.992. Number of larvae measured: 6 (0 mM), 7 (1 mM), 5 (2.5 mM), 6 (7.5 mM), 7 (15 mM), 3 (15 mM + VPA). Error bars are s.e.m. and where not visible are smaller than the symbol size. (**c**) Mean peak frequency as a function of PTZ concentration. The number of larvae is the same as in panel b.

We next characterized the response of zebrafish larvae to different PTZ concentrations. Figure 2a shows GCaMP6s recordings in 0 (control), 1, 2.5, 7.5 and 15 mM PTZ. The figure shows a clear dependence of both peak amplitudes and frequencies on PTZ concentration: at 1 and 2.5 mM PTZ, the peaks amplitudes are only slightly larger than control; on the other hand, at 7.5 and 15 mM PTZ we observed a marked increase in amplitude of the spikes and the appearance of regularly paced patterns of activity. Particularly at 15 mM PTZ the ΔF/F_0_ signal displayed long (about 5 minutes) and very large (ΔF/F_0_ up to 4–5) increases consisting of clusters of closely spaced spikes (Fig. 2a, inset). Each burst is followed by a period of low activity before a new burst appears. We observed some variability in the latency for appearance of these characteristic convulsive clusters of seizures (as visible from a comparison between Fig. 1b and the trace at 15 mM in Fig. 2a), but the periodicity of the convulsive and rest periods was remarkably reproducible across larvae.

Observation of the data in Fig. 2a shows that GCaMP measurements are sensitive to a range of activity alterations as induced by different PTZ concentrations. Our recordings enabled quantitative analysis of calcium activity in terms of both amplitude and frequency. Based on automated data analysis (see Methods) we identified each peak and measured its amplitude and the frequency of firing in the recordings. Figure 2b-c show the peak amplitudes and frequencies measured at different PTZ concentrations in the range between 0 and 15 mM. The ΔF/F_0_ peak fluorescence (Fig. 2b) exhibits a linear dependence on PTZ concentration (R = 0.992). Firing rates (Fig. 2c), on the other hand, do increase with PTZ concentration but in a nonlinear manner. In the figures, measurements are also reported for larvae that had been pre-incubated with Valproic Acid (VPA, 5 mM) for one hour before the treatment with PTZ (at 15 mM). The data clearly show that the anti-epileptic effect of this drug (one of the most commonly used in the treatment of epilepsy in humans) was measured extremely well by our method, since the ΔF/F_0_ peak amplitude and frequencies measured upon treatment with this drug were not distinguishable from the control levels. The linear response observed for the ΔF/F_0_ signal over the range of PTZ concentrations explored is interesting in the frame of studying mutations, which have been associated with epilepsy but may not lead by themselves to evident behavioural phenotypes. More generally, the method lends itself to the characterization of sub-threshold alterations of activity, due to genetic mutations or pharmacological treatments. Using PTZ as a standard in the field, we assessed the simplest and most robust method to quantify the effects of this compound on larval brain activity. For application of GCaMP6s activity measurements in a high-throughput assay (see below) we tested whether a simple time-averaged fluorescence over the whole recording in a given condition would be effective in detecting alterations of brain activity in a concentration-dependent manner. The data are shown in Supplementary Figure S1 and demonstrate that this simpler method is applicable to an analysis requiring less detail but faster processing such as demanded in high-throughput assays.

### Correlation analysis among brain regions and muscle movements

**Figure 3:**
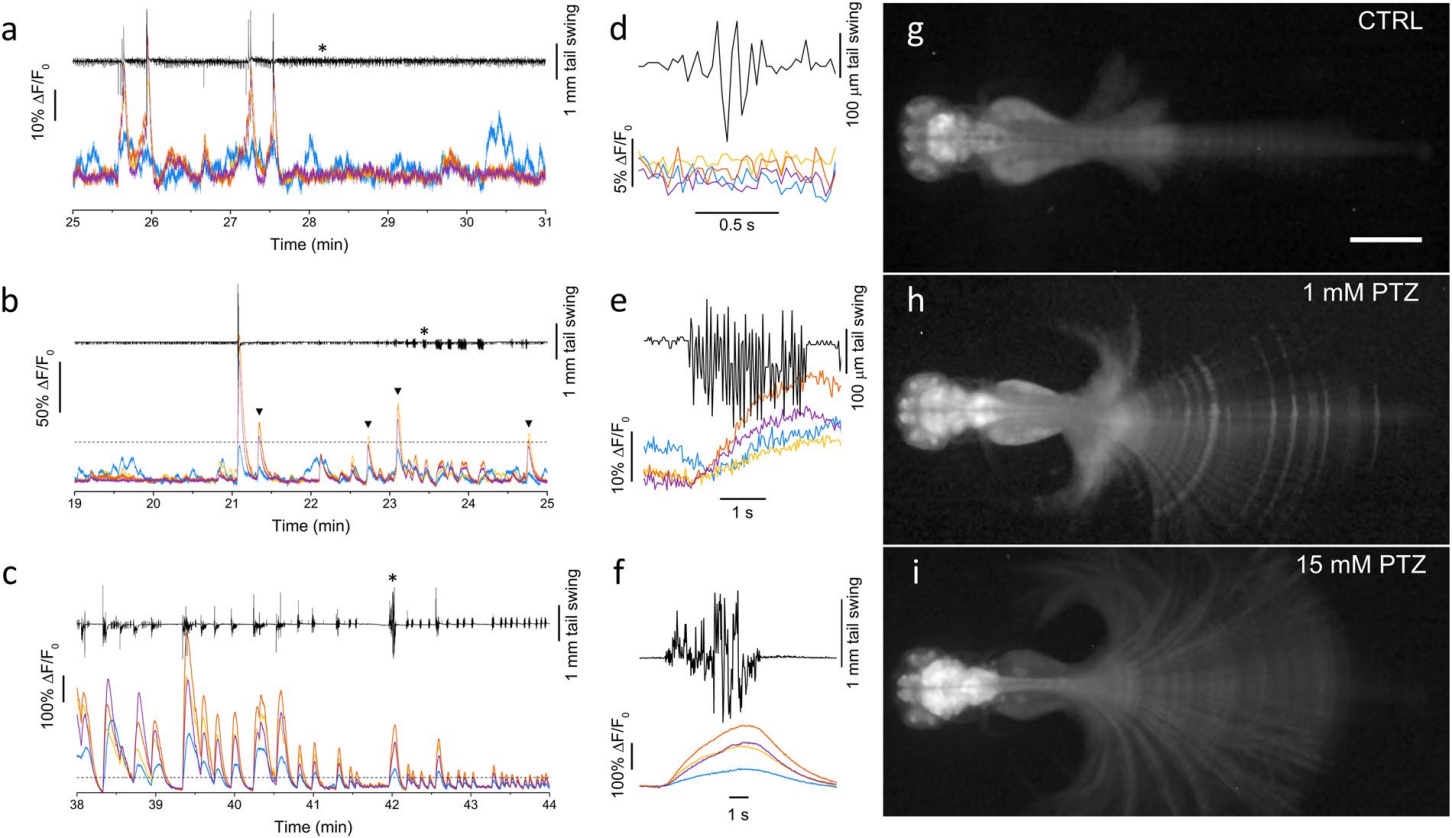
Simultaneous measurement of brain activity and tail movement. (**a**) Time trace of tail movement (black line) and fluorescence (ΔF/F_0_) analysed in control conditions (0 mM PTZ) for four brain regions color-coded as in Fig. 1a. The traces show correlations between fluorescence peaks and tail movements for optic tectum, cerebellum and medulla, while the telencephalon exhibits some degree of activity not correlated with tail movement. (**b-c**) Tail movement and brain activity analysed as in panel a at 1 and 15 mM PTZ, respectively. The dashed lines show a ΔF/F_0_ level of 0.4 corresponding to the highest peaks typically measured in control conditions. The arrowheads in panel b highlight peaks overcoming the 0.4 level and not corresponding to large tail movements. (**d-e-f**) Expanded time scale traces of the fragment indicated by the asterisk in a, b and c, respectively. Each panel shows movement and fluorescence traces (color-coded as in panels a-c) corresponding to a typical tail movement observed in the corresponding condition. (**g-h-i**) Fluorescence images of head-restrained larvae (see Methods) in 0, 1 and 15 mM PTZ. Images are maximum intensity projections over a time interval of 10 min, chosen during typical locomotor activity of the tested condition. Panels h and i are displayed with gamma = 0.4 to avoid saturation in the encephalon and still have good contrast on the tail. Scale bar 500 µm.

One of the main advantages of optical imaging with respect to the use of an electrode is the capability of simultaneously monitoring activity in different brain regions. Furthermore, with the very large field of view of our microscope we could image the whole larva, thus conducting simultaneous measurements of brain activity and tail movement (see Supplementary movie 1; the tail was free to move due to the method adopted for mounting the larva on the slide, as described in Methods). Imaging of the tail and its movements was facilitated by the fluorescence signal from GCaMP6s expressed in neurons of the spinal cord. We analysed fluorescence dynamics in four distinct brain regions: telencephalon, optic tectum, cerebellum and medulla. Figure 3 shows typical results obtained for the recording of brain activity and tail movement at 0, 1 and 15 mM PTZ concentration. The general patterns of activity and peak amplitudes reflect the results shown in Fig. 2 for the signal averaged over the whole brain. Distinguishing the different brain regions, however, allows some interesting observations. During spontaneous activity (CTRL, Fig. 3a) we noticed that the activities in the optic tectum, cerebellum and medulla were well correlated, while the telencephalon exhibits independent patterns. Moreover, while the larger peaks in the three caudal regions of the brain correlated with large movements of the tail, telencephalon activity was not always linked with large movements. Figure 3d shows an enlarged part of the fluorescence and tail movement traces, demonstrating that in this condition most of the tail movements are brief, small amplitude beats consisting of a single small tail swing. These movements (also shown in Fig. 3g) can be classified as “beat and glide” movements^27^, and are not paired with measurable changes of fluorescence (most likely due to the loss of sensitivity caused by integrating the signal over large encephalic areas in wide field imaging). Compared to control, at 1 mM PTZ (Fig. 3b) we observed that telencephalon correlation with the caudal regions increased, even though some instances of independent peaks persisted. As shown also by the data in Fig. 2, at this PTZ concentration, we observed the appearance of some peaks with a larger amplitude than control peaks (the dotted line indicates the maximum value of ΔF/F_0_ typically reached in control recordings). These peaks can therefore be attributed to the alterations induced by PTZ. It is interesting to notice that, some of these peaks were associated with large tail movements (that would be conducive to swimming, if the larva were free to swim), but many others (highlighted by arrowheads in the figure) were not associated with any tail movement or, at most, with very small tail deflections that would not produce detectable movements in behavioural assays. Indeed we noticed that additionally to the infrequent but large tail swings (Fig. 3b,h), at 1 mM PTZ concentration, the larva exhibited frequent small tail movements associated with detectable changes in the ΔF/F_0_ signal (Fig. 3e). We noticed that, compared to control movements, these are more clustered and exhibit a larger number of tail beats per event. These movements can thus be classified as “dart” movements^27^. At 15 mM PTZ (Fig. 3c) we observed the complete synchronicity of activity in the four brain regions, the appearance of very large, regularly paced peaks of activity which are associated with larger tail movements, i.e. with the stage III^13^ convulsive swimming detected also in behavioural assays. We also noticed a correlation between peak intensity and the amplitude of tail movement, with higher peaks being associated with powerful swimming bouts (Fig. 3c,f,i) and lower peaks with smaller tail fluctuations. Supplementary movies 2–4 show examples of the three types of tail movements described in the three conditions tested. Despite vigorous tail movements, the agarose-embedded head of the larva remained very stable during the measurements, thus avoiding potential movement artefacts (see methods for more details). The stability of the sample can be appreciated in the Supplementary movies 1–4, enabling measurements in the absence of paralyzing agents, with the advantage of allowing combined fluorescence and tail movement measurements such as those shown in Fig. 3. Supplementary Fig. S2 shows ΔF/F_0_ traces obtained after paralyzing the larva with d-tubocurarine, demonstrating the same features shown by the data in the absence of paralyzing agent (Fig. 3).

**Figure 4:**
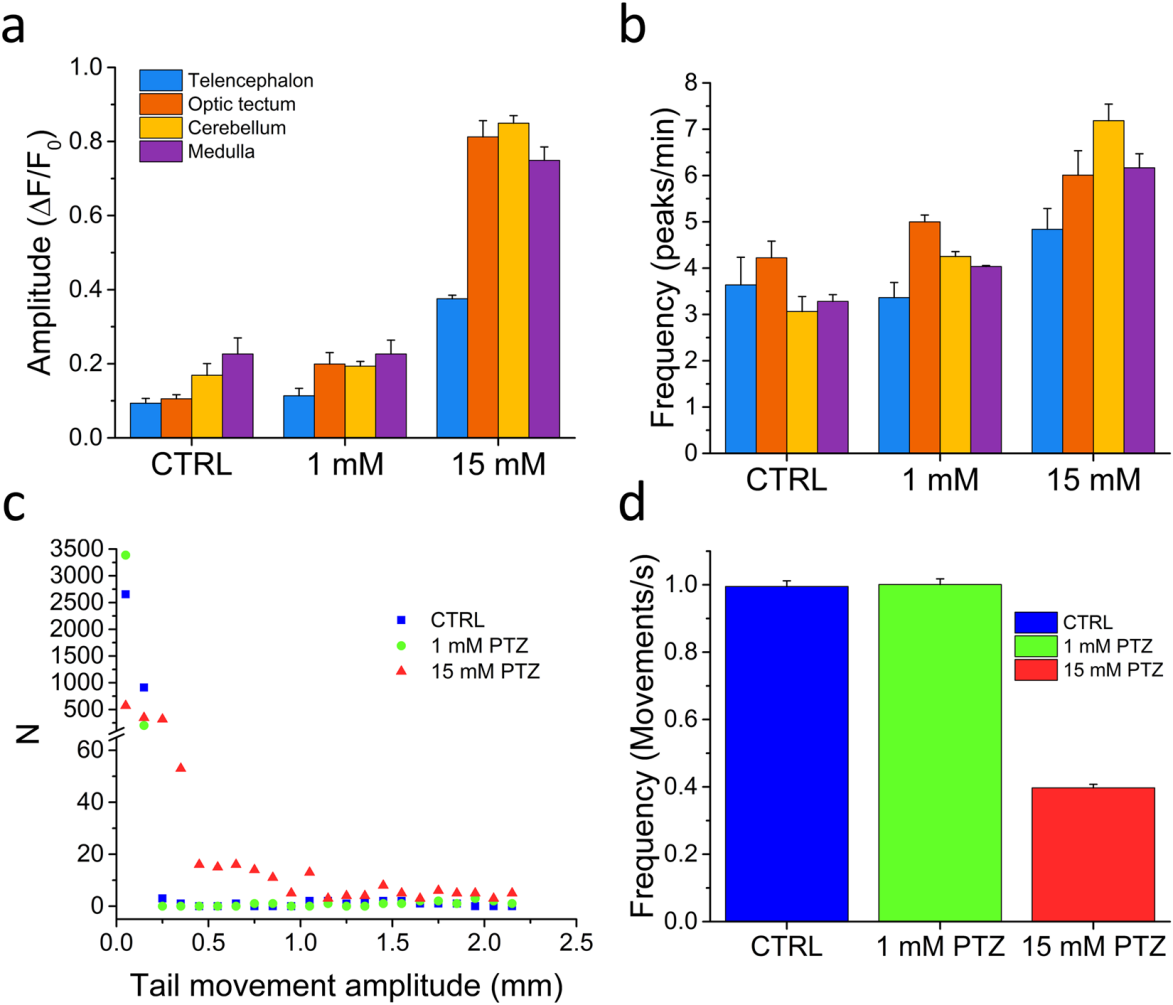
Distributions of brain activity and tail movement. (**a-b**) Bars represent mean peak amplitude and mean peak frequency, respectively, in the four brain regions highlighted in Fig. 1a, for the three different conditions tested (0, 1 and 15 mM PTZ). Error bars: s.e.m. Number of larvae measured: 3 for each experimental condition. (**c-d**) Distribution of tail movement amplitude and mean frequency for the three experimental conditions tested (one larva per condition). Error bars in d represent the uncertainty of a Poisson’s distribution.

We next analysed the amplitudes and frequencies of both calcium and tail signals. Figure 4a-b show the mean peak amplitude and firing rate, respectively, measured in the different brain regions at 0, 1 and 15 mM PTZ. We can observe that during spontaneous activity cerebellum and medulla exhibit slightly larger peaks, while firing rates are quite similar across the brain, with the optic tectum having a slightly higher rate than cerebellum and medulla. The introduction of PTZ, on the other hand, induced a concentration-dependent imbalance of the caudal regions (optic tectum, cerebellum and medulla) compared to telencephalon. The imbalance was evident both in terms of amplitudes (with a very large increase at 15 mM PTZ) and rates. Fig. 4c-d show the effects of PTZ on tail movement. Overall, the distributions of tail movement amplitudes (Fig. 4c) demonstrate that PTZ most evidently induced seizures at 15 mM concentration, characterized by large tail swings (with amplitudes larger than 0,4 mm and up to 2.2 mm on our scale, where neither control nor 1 mM PTZ exhibited any event), These convulsive bursts are followed by a static phase, accounting for the lower average tail beat frequency in 15 mM PTZ compared to control and 1 mM (Fig. 4d).

**Figure 5:**
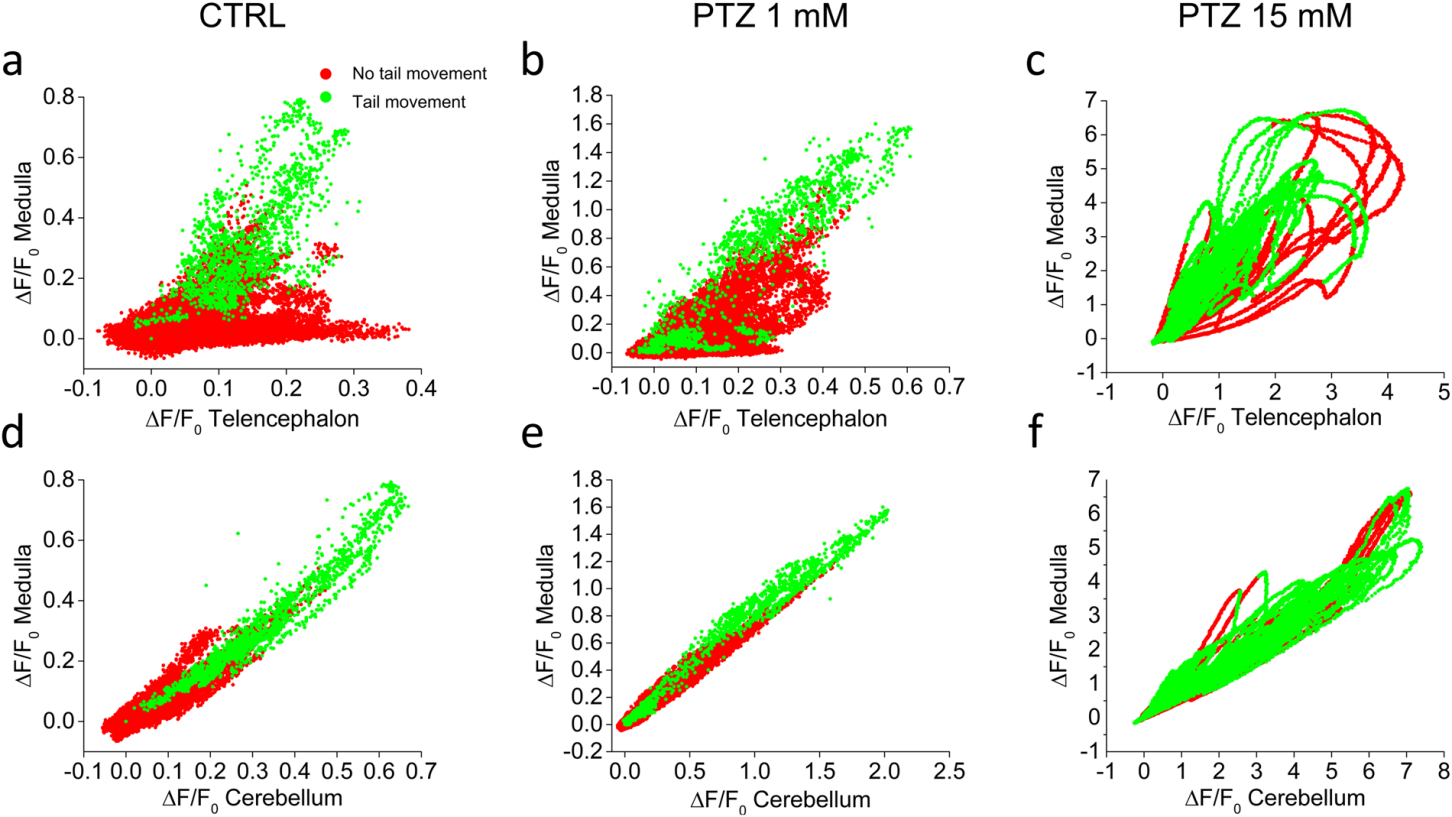
Correlations of brain activity in different regions with locomotor activity. Correlation plots of ΔF/F_0_ for chosen pairs of brain regions in control, 1 and 15 mM PTZ. Correlations a﻿re shown between telencephalon and medulla (panels a-c) and between cerebellum and medulla (panels d-f). Red and green symbols represent fluorescence data corresponding to resting and swimming times, respectively (see Methods). In panels a and b it is evident the presence of a subset of data characterized by telencephalon activity not associated with medulla activity (panel a) or associated with only low levels of medulla activity (panel b); all those data points correspond to time intervals in which no tail movement takes place. Measurements shown are from the recording performed on one larva.

We then analysed the correlations between neuronal activities in different brain regions and with tail movement. We first considered the correlation between telencephalon and medulla, which was observed to be low in control condition and low PTZ concentrations (see Fig. 3). Fig. 5a shows correlation in one typical recording performed without PTZ. The data confirmed the presence of a significant fraction of telencephalon activity not correlated with medulla, as quantitatively demonstrated also by the low correlation coefficient (R = 0.35). In order to assess the correlation of any brain activity with tail movement, we identified points in the fluorescence trace occurring during movement and plotted them with green symbols in the figure, while red symbols represent brain activity not associated with movement. We noticed that the independent telencephalon activity was not associated with any tail movement, while neuronal spiking occurring in the telencephalon synchronously with that of medulla was associated with locomotor activity. Figure 5b-c show the same measurement performed at 1 and 15 mM PTZ, respectively. The data show that, with increasing PTZ concentrations, there was a progressive reduction of the fraction of independent telencephalon activity, which was measured by an increasing correlation coefficient (R = 0.61 and R = 0.89 at 1 and 15 mM PTZ, respectively). At 1 mM PTZ, a population of data points corresponding to medium levels of correlation were still visible and maintained the property of spontaneous telencephalon activity, i.e. they were not functionally linked with tail movement. At 15 mM PTZ, on the other hand, the correlation between telencephalon and medulla was high and most of the neuronal activity was associated with tail movement. Figure 5d-f show correlation measurements with tail movement analysis for cerebellum and medulla. In this case, we could observe complete correlation of the two areas in all conditions (R = 0.94, 0.98 and 0.98 at 0, 1 and 15 mM PTZ, respectively) and a tight link between activity in these areas and locomotor activity.

A systematic characterization of the correlation of activity across all the four brain areas (for left and right hemisphere, Supplementary Fig. S3) confirmed the observation that increasing PTZ concentrations induce seizures consisting of highly correlated activity across the whole brain. At low (1 mM) PTZ concentration and in control conditions, on the other hand, we could observe the progressive loss of correlation along the rostro-caudal axis, with the telencephalon being the region which less correlates with the others. In all conditions, we observed a high correlation between left and right side of each region. We also observed that after onset of PTZ effect, correlations maintained their characteristic patterns for the whole duration of our measurements. These results are in line with previously observed whole-brain activation induced by PTZ^28, 29^ and with the common use of PTZ at 10–15 mM concentration to induce generalized seizures in the larva.

### High-throughput combined fluorescence and behavioural recordings

**Figure 6:**
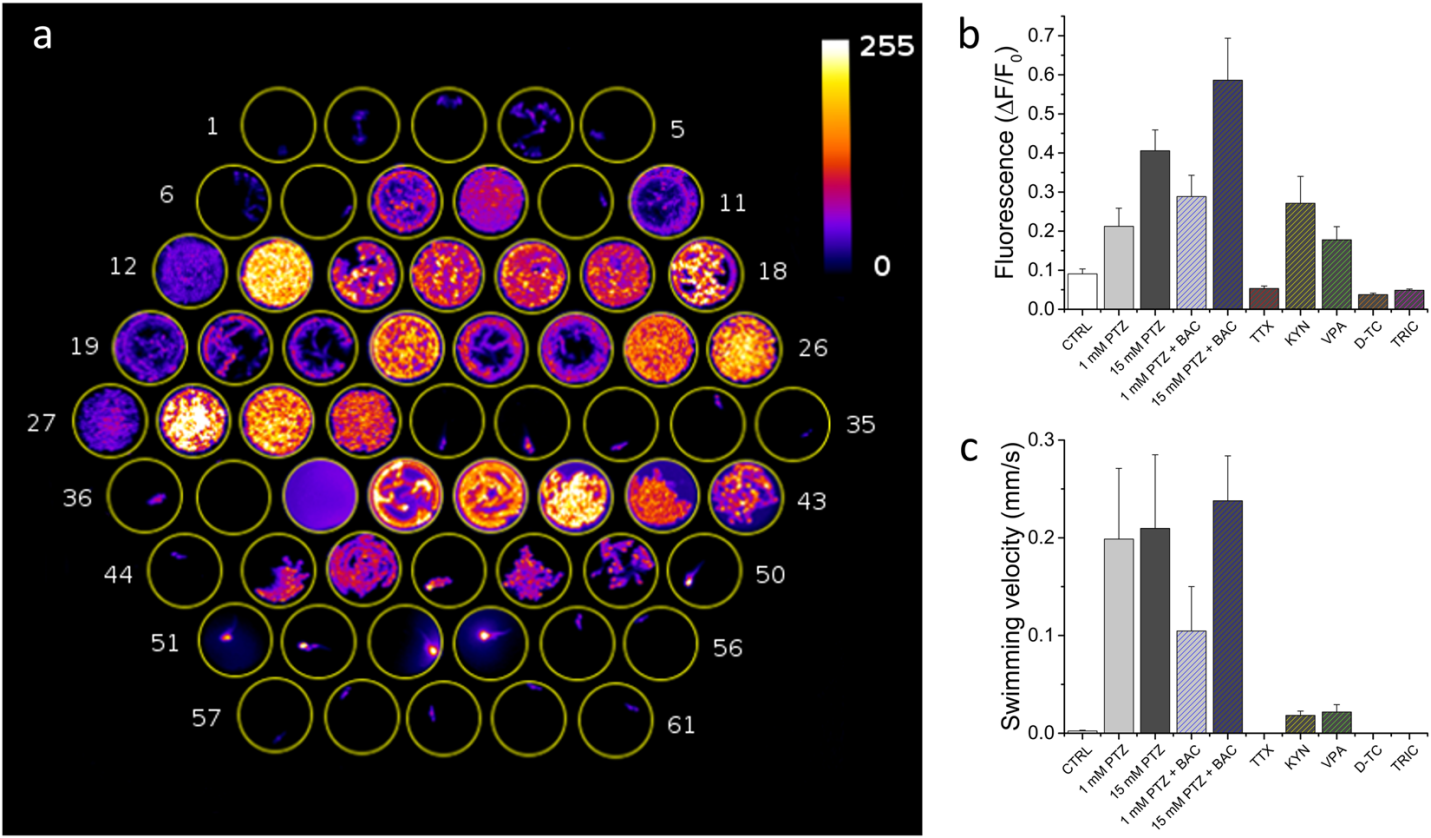
High-throughput combined fluorescence and behavioural assay. (**a**) Maximum intensity projection of a 13 minute segment of the recording. Well content: 1–6 control, 7–12 1 mM PTZ, 13–18 15 mM PTZ, 19–24 1 mM PTZ + 50 μM Baclofen, 25–30 15 mM PTZ + 50 μM Baclofen, 31–36 15 mM PTZ + 5 μM Tetrodotoxin, 37 fish water, 38 fish water + 2 mM Kynurenate, 39–43 15 mM PTZ + 2 mM kynurenate, 44–49 15 mM PTZ + 5 mM Valproate, 50–55 15 mM PTZ (after pre-incubation in 2 mM d-tubocurarine for 10 minutes before starting the measurement), 56–61 15 mM PTZ + 160 mg/L Tricaine. (**b**) Average ΔF/F_0_ and (**c**) swimming velocity for the different conditions tested. Error bars: s.e.m.

One of the main advantages of zebrafish is the possibility of performing high-throughput drug screenings. The data shown in Figs 2–3 demonstrate that measurements of neuronal activity can complement behavioural measurements by providing information on activity alterations not always linked to altered swimming behaviour of the larva. To this purpose, we implemented fluorescence measurements in a wide-field configuration at low magnification, enabling imaging over a large area (~50 cm^2^). With this optical system, we could simultaneously measure GCaMP6s fluorescence and larva position from 61 larvae in parallel, testing ten different experimental conditions with statistics of 5–6 larvae per condition. We chose to reproduce some of the conditions tested in the paper (i.e. 0, 1 and 15 mM PTZ treatment, Valproate, Tricaine and TTX treatments in the presence of 15 mM PTZ), together with a test on the effects of two compounds acting on synaptic transmission and previously shown to alter PTZ-induced seizures as measured with electrical recordings^13^: kynurenate (a non-specific blocker of postsynaptic glutamate receptors, which was shown to reduce seizure amplitudes) and Baclofen (a GABA_B_ receptor agonist, which was shown to increase the amplitude of seizures). Supplementary movie 5 shows a 13 minutes segment of the recording. For an overall view of larva behaviour and fluorescence in each well, we produced a maximum intensity projection (MIP) of 45 minutes (Fig. 6a), after one hour of drugs exposure. The figure immediately demonstrates the effects of increasing PTZ concentrations, as visible from an increased motility of the larvae at 1 mM PTZ (wells 7–12) compared to control (wells 1–6) and a further increase of motility, associated with a very large increase of fluorescence at 15 mM PTZ (wells 13–18). Introduction of Baclofen potentiated the fluorescence signal both at 1 (wells 19–24) and 15 mM PTZ (25–30). Tetrodotoxin (wells 31–36), on the other hand, completely suppressed neuronal activity, even in the presence of 15 mM PTZ, as show in Fig. 1d, with consequent decrease of signal and larva paralysis. The effect of kynurenate (wells 39–43) was not apparent in the MIP analysis but became more evident in a subsequent quantitative analysis of ΔF/F_0_ and movement (Fig. 6b-c). An important point to note in this type of assay is that some compounds (for example kynurenate) might display significant fluorescence at the wavelengths employed for GCaMP6s excitation and detection, as demonstrated by the background measured in the well containing only kynurenate (well 38) compared to fish water (well 37). Valproate showed its effectiveness in the MIP measurement (wells 44–49) where it decreased both larva motility and fluorescence in the presence of 15 mM PTZ. D-tubocurarine (wells 50–55) caused larva paralysis, but unfortunately often caused the death of the larvae at the concentration used. Finally, tricaine (wells 56–61) showed its anaesthetic effect in terms of both reduced fluorescence and motility of the larvae. The results of the high-throughput assay were quantified also by measuring the average ΔF/F_0_ (Fig. 6b) and larva swimming velocity (Fig. 6c) in all conditions tested. Velocities were calculated from trajectories shown in Supplementary Fig. S4.

## Discussion

The development of new AEDs, capable of successfully treating the relevant fraction (about 35%) of patients not responsive to currently available drugs, would highly benefit from the possibility of performing high-throughput screenings and drug refinement on animal models accurately portraying the molecular framework of specific epileptogenic processes in those patients. In this regard, zebrafish represents, for many reasons, an ideal model to pursue this goal. It is a vertebrate model with sufficient similarity in its brain architecture and neurochemistry to allow comparisons with the human brain^11^, thus enabling a study of seizure dynamics in different regions and on different neuronal populations. Moreover, the zebrafish genome is characterized by the presence of orthologues of at least 70% of all human genes and is easy to be genetically manipulated for the production of transgenic lines reproducing mutations found to be linked with epilepsy in humans^10^. These features have made this animal model very prominent in recent years for the study of epilepsy and for the screening and development of new AEDs^16^. Current approaches are mainly based on electrophysiological measurements and behavioural assays. The former are very informative on overall brain activity but lack spatial discrimination, thus failing to provide detailed information on the localization of the epileptic foci and the dynamics of propagation of seizures. Recently a multi-electrode system has been developed capable of addressing long-term electrical measurements in parallel on several zebrafish larvae and providing independent measurements of electrical activity in up to five regions of the larva encephalon along the rostro-caudal axis^30^. Behavioural assays, on the other hand, are very simple to conduct and easily lend themselves to high-throughput screenings, and have been, indeed, successfully used to identify new potential AEDs^20, 31, 32^. These assays rely on the detection of large swimming bouts and locomotor alterations in zebrafish larvae induced by either specific mutations (for example mind bomb^33^) or chemical treatments (typically PTZ) and screening for compounds capable of rescuing normal locomotor phenotype. Although quite simple and effective, we argue that this method may suffer from two limitations. Firstly, it detects only significant locomotor seizures and might miss sub-threshold or pre-epileptic events; secondly, it provides a positive cue for compounds that may have, for example, a strong anaesthetic or paralyzing effect and would, therefore, be undesirable as anti-epileptic therapeutic agents.

To overcome the limitations of current methods, here we describe an approach based on employing a transgenic line expressing GCaMP6s in all neurons and directly measuring neuronal activity in larvae treated with PTZ. This compound was chosen since it has been widely proven to be a good way to induce convulsions in zebrafish that are considered a good model for epileptic seizures in humans. The data in Figs 1 and 2 demonstrate that fluorescence recordings show characteristic changes in ΔF/F_0_ peaks with increasing amplitudes for increasing PTZ concentrations in the range tested. We also showed that the simple measurement of the average ΔF/F_0_ signal integrated over time and over the whole larva brain is sufficient to detect alterations induced by PTZ even at very low (1 mM) concentrations. A full characterization of PTZ effects on zebrafish larvae has demonstrated a concentration-dependent variety of behaviours classified in different stages^13^: an increase of swimming activity with respect to control (stage I), whirlpool-like circular swimming (stage II) and clonus-like convulsions with loss of posture (stage III). The PTZ concentrations normally employed to induce generalized seizures in zebrafish larvae are in the range of 10–15 mM. It has been shown^13^ that lower concentrations delay the onset of seizures and, below a given threshold, for example at 2.5 mM, full convulsions (stage III) are never reached, while stages I and II can still be observed. Thus, these low PTZ concentrations can be used to induce a mild or pre-epileptic state in the zebrafish larva. This sub-threshold state may also mimic the effects induced by genetic alterations not capable by themselves to induce seizures but contributing to determine a genetic epileptogenic background. In our work, it is interesting to notice that, in the presence of 1 mM PTZ, some fluorescence spikes could be observed with amplitudes higher than the typical maximum amplitude of control measurements, as shown by the dotted line in Fig. 3. Some of these peaks (highlighted by the arrowheads in Fig. 3c) were not conducive to tail movement or only caused very small oscillations that would not lead to a locomotion detectable in behavioural assays. Thus, it is evident that the direct measurement of brain activity represents a powerful means for characterizing sub-threshold alterations. This sensitivity is essential to dissect contributions of individual mutations and develop specific drugs for each of them and, therefore, to test drugs with much higher effectiveness.

Compared to electrophysiology, optical measurements have the advantage to be non-invasive and to provide full mapping of brain activity. Previously, PTZ was investigated with organic calcium indicators and two-photon microscopy in *Xenopus laevis* tadpoles to measure neuronal activity at single cell level^34^. A genetically-encoded FRET-based calcium indicator was first applied in zebrafish by Tao *et al*. for the study of PTZ-induced seizure propagation in the larva brain^35^. Recently GCaMP-expressing zebrafish larvae were used in a microfluidic system with two-photon excitation to measure neuronal and locomotor response to chemical stimuli^36^.

The measurements shown in Figs 1b and 2a lend themselves to a direct comparison between optical and electrophysiological measurements. The regularly-paced large ΔF/F_0_ peaks seen during seizures at 15 mM PTZ (Fig. 2a) were remarkably similar to the results obtained with electrical measurements^30^ and to the envelope of the data measured with the FRET-based genetically encoded calcium indicator^35^. Moreover, the data shown in Fig. 2c for spiking frequencies compares well with data published at the same PTZ concentration with electrophysiological recordings^13^. We also performed a direct comparison between optical and electrophysiological measurements by simultaneously recording GCaMP6s fluorescence and electrical activity with an extracellular electrode placed in the optic tectum. The data shown in Supplementary Fig. S5 demonstrate an excellent sensitivity of the ΔF/F_0_ signal averaged over the whole larva brain with the tectal field recording. It can be noticed that the fluorescence signal monitors both ictal and inter-ictal activity. The correspondence between our optical measurements and the data obtained with electrophysiological methods confirms the effectiveness of calcium measurements for monitoring seizures in zebrafish larvae, at least in a time domain compatible with the dynamics of available genetically-encoded calcium indicators.

In this study, the choice of wide-field over confocal or two-photon imaging is due to the time resolution we wanted to achieve in calcium imaging, in order to detect the fast dynamics of calcium peaks in the whole brain. Moreover, the large field of view allowed simultaneous measurements of brain activity and tail movement. However, implementing fast volumetric imaging methods^24^ will allow extending this study down to the cellular level, without significant loss in temporal resolution. We thus consider the demonstration conducted in this study as the basis for complementing electrophysiological information with optical measurements, providing more detailed data on activity mapped at single brain region level (Figs 3–5) and, in the future, down to single neurons, enabling a better understanding of seizure onset and propagation.

The simplicity and speed of wide-field imaging comes with loss of the optical sectioning capability of the larva brain, as would be attainable with confocal microscopy. This might especially affect the attribution of fluorescence coming from a given region in the image plane to specific brain structures, which are located at different depths within the brain. Considering the projection of the whole brain of the larva onto a single plane in wide field imaging, we assessed potential bleed-through of spurious fluorescence from adjacent regions (especially for deeper structures) due to photon scattering in the brain volume. Supplementary Figure S6 shows this characterization, demonstrating that all features and details of the larva encephalon as observed in the confocal image are well visible also in the wide field image. Moreover, we find that cross-talk caused a deviation of measured fluorescence by less than 10% from ideal measurements. We notice that in any case, based on the data shown in Supplementary Figure S6, this small amount of possible cross-talk only regards optic tectum and telencephalon, as expected from their relative position and 3D structure; however, our data (Fig. 3a) demonstrate true uncorrelated activity of telencephalon without significant cross-talk of this signal with the optic tectum.

Another significant advantage of optical measurements is the ease of implementation of high-throughput screening methods^37^ in which brain activity is measured in an array of larvae undergoing different treatments. Here we present a demonstration of high-throughput measurement of fluorescence and motility of 61 larvae undergoing a battery of ten different conditions. Considering the resolution of the optical system (sufficient to image the whole larva and track it adequately for behavioural measurements), the sCMOS detector area and the LED power, it is easy to imagine a scalability of this measurement to a much larger number of wells beyond the proof-of-principle shown here.

Moreover, optical measurements can be combined with other techniques (including electrophysiological, behavioural assays, optogenetic neuronal stimulation and/or inhibition) to facilitate a more extensive and powerful study of epileptic seizures in zebrafish and expand the potential of this model for the comprehension of this pathology in its diverse genetic variants as well as for the screening and development of new AEDs.

Finally, the method (and its high-throughput drug screening applications) lends itself to the study of neuronal activity alterations in any other neuropathology for which a genetic or pharmacological model is available in zebrafish.

## Materials and Methods

### Zebrafish transgenesis and maintenance

We generated a zebrafish Tg(elavl3:GCaMP6s) line using a Tol2 construct with elavl3 promoter that drives the expression of the genetically encoded calcium indicator GCaMP6s in all neurons^26^. One-cell stage zebrafish embryos were injected with the Tol2 transgene construct together with the transposase mRNA. Mosaic transgenic fish displaying a strong fluorescence at 24 hpf were selected and raised to adulthood. Offspring from one single selected founder was finally used to establish a new stable transgenic line used for the experiments.

Zebrafish of the Tg(elavl3:GCaMP6s) strain were maintained according to standard procedures^38^. Larvae were raised in 0.003% N-phenylthiourea (P7629, Sigma) to inhibit melanogenesis, avoiding the formation of skin pigments.

The Tol2-elavl3-GCaMP6s plasmid was a gift from Dr. Misha Ahrens (Addgene plasmid # 59531)

All experiments were carried in accordance to Italian law on animal experimentation (D.L. 4 March 2014, n.26), under authorization n. 407/2015-PR from the Italian Ministry of Health.

#### Zebrafish larvae preparation

Each 4 dpf Tg(elavl3:GCaMP6s) zebrafish larva was transferred into a reaction tube containing 1.5% (w/v) low gelling temperature agarose (A9414, Sigma) in fish water (150 mg/L Instant Ocean, 6.9 mg/L NaH_2_PO_4_, 12.5 mg/L Na_2_HPO_4_, pH 7.2), kept at 38 °C. The larva was then taken up with a syringe in a glass capillary (O.D. 1.5 mm); after gel polymerization, the agarose cylinder was gently extruded from the capillary and laid on a microscope slide. In this phase, care was taken to orient the embedded larva with its dorsal portion facing upwards. A small drop of melted agarose was then placed on the head portion of the larva in order to immobilize the head in the precise orientation chosen. Next, the agarose cylinder below the yolk was cut off with a scalpel and removed, in order to free the tail. This procedure not only allows measurements of tail movement but also improves the quality of encephalic imaging by preventing vibrations otherwise transmitted through the agarose cylinder (see Supplementary movies 6–8, showing that movements associated with convulsions, visible in the fully embedded larva, are drastically reduced by freeing the tail, almost as effectively as with a paralysing agent). A flow chamber was then assembled by attaching a coverslip to the microscope slide with double-sided tape (1.5 mm thick). In every experiment the chamber was initially filled with fish water.

To perform high-throughput measurements we designed a black PVC multi-well plate composed of 61 cylindrical wells (diameter: 7 mm; depth: 5 mm; volume: ~200 μL) arranged in an hexagonal geometry. The material chosen to build the multi-well plate had the advantage of both being inert and offering a dark background for fluorescence measurements. Each 4 dpf Tg(elavl3:GCaMP6s) zebrafish larva was transferred into one of the 61 wells with an appropriate volume of fish water. After 20 min of habituation, 10 min of control recording were performed and then drug stock solutions were added to each of the 61 wells to obtain the desired final concentrations in 180 μL final total volume. Measurements were then run for 2 hours, a time sufficient for all the tested drugs to exert their action.

#### Chemicals

Stock solutions of pentylentetrazole (PTZ; P6500, Sigma-Aldrich), valproic acid (VPA, P4543, Sigma-Aldrich), tricaine (A5040, Sigma-Aldrich), kynurenic acid (KYN, ab120256, Abcam), tetrodotoxin (TTX) and Baclofen (BAC) were prepared by dissolving in milliQ water (at 100 mM concentration for PTZ, VPA and KYN; 4 g/L for tricaine; 2 mM for TTX; 10 mM for Baclofen;). The final concentrations used in the experiments were obtained by diluting each stock in fish water. D-tubocurarine (D-TC; 93750, Sigma-Aldrich) was dissolved at 2 mM concentration directly in fish water and used by pre-incubating the larvae for 10 min before measurements.

#### Wide-field fluorescence microscope

Imaging of the mounted larvae was performed using a custom-made upright fluorescence microscope. We imaged either the entire zebrafish larva (using EC Plan-NEOFLUAR 2.5x/0.085 M27, Carl Zeiss Microscopy) or only the encephalic region (with EC Plan-NEOFLUAR 5x/0.16 M27, Carl Zeiss Microscopy). Fluorescence was excited in wide-field configuration using an LED with emission centred at 470 nm (M470L3, Thorlabs), followed by an excitation band-pass filter (469/35 nm, FF01–469/35, Semrock). The epifluorescence detection was implemented with a dichroic mirror (DC FF495-DI02, Semrock) and an emission band-pass filter (525/39 nm, MF525-39, Thorlabs). The fluorescence signal was recorded with a sCMOS camera (OrcaFLASH 4.0, Hamamatsu Photonics) with 16-bit dynamic range.

To perform high-throughput measurements the excitation LED was equipped with a diffuser to produce an even illumination over an area of about 50 cm^2^, covering all the multi-well plate. In order to image such a large area, a wide-angle lens (MVL8M23, Thorlabs) with 8 mm focal length was mounted directly on the camera.

#### Brain activity recordings

Imaging was performed with the camera operating at 5 Hz (200 ms exposure time); each measurement was composed of a 10-minute control recording (during which the larva was kept in fish water), followed by addition of the drug solutions at the desired concentration and subsequent recording for 30 to 120 minutes.

When performing measurements on the whole larva to obtain data on tail movement along with fluorescence, the camera was operated at 33 Hz frame rate with 5 ms exposure time (chosen to guarantee sharp images of the tail even during fast movements).

High-throughput measurements were performed operating the camera at 5 Hz (200 ms exposure time).

#### Data analysis

In each wide-field experiment, the GCaMP6s fluorescence signal from the whole larval encephalon or from different brain regions (telencephalon, optic tectum, cerebellum, medulla) was measured using ImageJ, by manually selecting the desired region of interest (ROI). The ROI’s mean fluorescence intensity for each time point was then calculated and, after background subtraction, the values obtained were further processed using a custom Matlab script to obtain the ΔF/F_0_ signal (following ref^39^) as described below.

The baseline for every time point (F_0_) was calculated (using Matlab function *msbackadj* with a running window and a step size of 2000 and 13333 points for 5 Hz and 33 Hz recordings, respectively) and subtracted from the fluorescence signal (F), thus producing ΔF. These values were then normalized by dividing them by the baseline itself, obtaining ΔF/F_0_.

The traces shown in Fig. 1b-c were processed differently, to highlight the dynamics of the basal brain activity in the experimental conditions shown. The mean fluorescence intensity of each 10 min control recording was calculated and used as F_0_ in the ΔF/F_0_ calculation of each trace. In this way, the trace was not flattened by running baseline subtraction.

Automatic peak detection was performed on ΔF/F_0_ traces using a custom Matlab script. The software first smoothed the traces by applying Savitzky-Golay filtering (polynomial order: 7; frame length: 31 for 5 Hz acquisitions and 207 for 33 Hz acquisitions) then ran *findpeaks* function with 0.02 ΔF/F_0_ minimum peak prominence as a threshold, to measure the number of peaks in the trace along with their maximum ΔF/F_0_ value. Tail movement analysis was first performed with ImageJ using a parabolic line selection with its focus centred on the tail position at rest. The intensity line profile of the selection was then measured in each frame and the coordinate of the maximum intensity point indicated the tail position at that time. Tail position values were then converted in mm.

On tail movement traces peak analysis was also performed, by setting the minimum peak prominence to 0.05 mm and the minimum peak distance to 0.3 s, as a threshold. These parameters were chosen to optimize true motion detection above noise.

Correlation plots shown in Fig. 5 were obtained by plotting against each other ΔF/F_0_ from two chosen regions. To distinguish fluorescence data occurring simultaneously with tail movement from those during rest, the tail position data were smoothed (running window of 150 points) and a threshold of 0.1 mm displacement of the tail was chosen to indicate significant movement.

Cross-correlation analysis shown in Fig. S1 was performed on brain recordings of larvae exposed to different PTZ concentrations, considering the four brain regions used throughout the paper and further dividing the data into the two hemispheres. For each condition considered, analysis was conducted on three progressive timeframes along a one-hour measurement. Correlation coefficients were calculated for any chosen pair of brain regions, averaged over three replicates and plotted in the cross-correlation matrix shown in the figure.

#### High-throughput analysis

The fluorescence recordings from the high-throughput measurements were analysed by selecting a region of interest (ROI) around each well and then running a custom ImageJ macro enabling parallel measurement of the maximum grey level detected in each ROI (after subtraction of the background measured in each ROI in a region of 230 px^2^ away from the larva). For larva tracking, the x,y coordinates of the centre of mass were extracted from the ROI after thresholding the image so to measure only pixels attributable to the larva. The routine ran these measurements on each frame of the acquired video. Subsequently, intensity data were analysed to obtain ΔF/F_0_ traces as described above, while coordinates data were used to plot the larva trajectory and calculate its speed for each pair of frames.

## Electronic supplementary material

Supplementary Information

Supplementary_Movie1_15mM_PTZ

Supplementary_Movie2_CTRL_beat&glide

Supplementary_Movie3_1mM_PTZ_Dart

Supplementary_Movie4_15mM_PTZ_seizure

Supplementary_Movie5_High-Throughput

Supplementary_Movie6_Agarose_embedded

Supplementary_Movie7_Tail_free

Supplementary_Movie8_Curarine
