# Supplementary material for: Optical mapping of neuronal activity during seizures in zebrafish
Source: Sci Rep. 2017 Jun 8;7:3025. doi: 10.1038/s41598-017-03087-z (PMC5465210; doi:10.1038/s41598-017-03087-z)
Supplement: Supplementary file 1 — Supplementary Information [file 41598_2017_3087_MOESM1_ESM.pdf]

SUPPLEMENTARY INFORMATION

Optical mapping of neuronal activity during seizures in zebrafish

L. Turrini[1], C. Fornetto[2], G. Marchetto[2], M.C. Müllenbroich[1,3], N. Tiso[4], A. Vettori[4], F. Resta[1], A. Masi[5], G..Mannaioni[5], F.S. Pavone[1,3,6], F. Vanzi[1,2,*]

[1]European Laboratory for Nonlinear Spectroscopy (LENS)
[2]Department of Biology, University of Florence, Italy
[3]Istituto Nazionale di Ottica (INO), CNR, Italy
[4]Department of Biology, University of Padova, Italy
[5]Department Neurofarba, University of Florence, Italy
[6]Department of Physics, University of Florence, Italy
[*]corresponding author (francesco.vanzi@unifi.it)

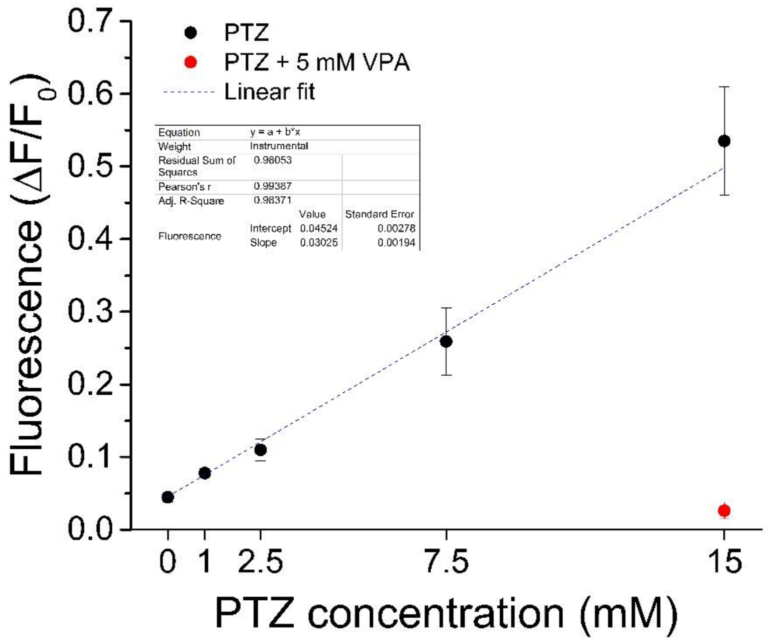

Figure S1. Effects of different PTZ concentrations on brain activity. Each symbol represents the mean change in fluorescence compared to baseline ($\Delta F/F_0$) of multiple larvae exposed to the same condition. Red symbol represents mean $\Delta F/F_0$ of larvae treated with 15 mM PTZ after a pre-incubation of one hour in 5 mM VPA. Dashed line is the linear regression through PTZ-only points. Error bars: SEM. Errors of the points at 0 and 1 mM PTZ are smaller than symbols size. Number of larvae measured: 6 (0 mM), 7 (1 mM), 5 (2.5 mM), 6 (7.5 mM), 7 (15 mM), 3 (15mM + VPA).

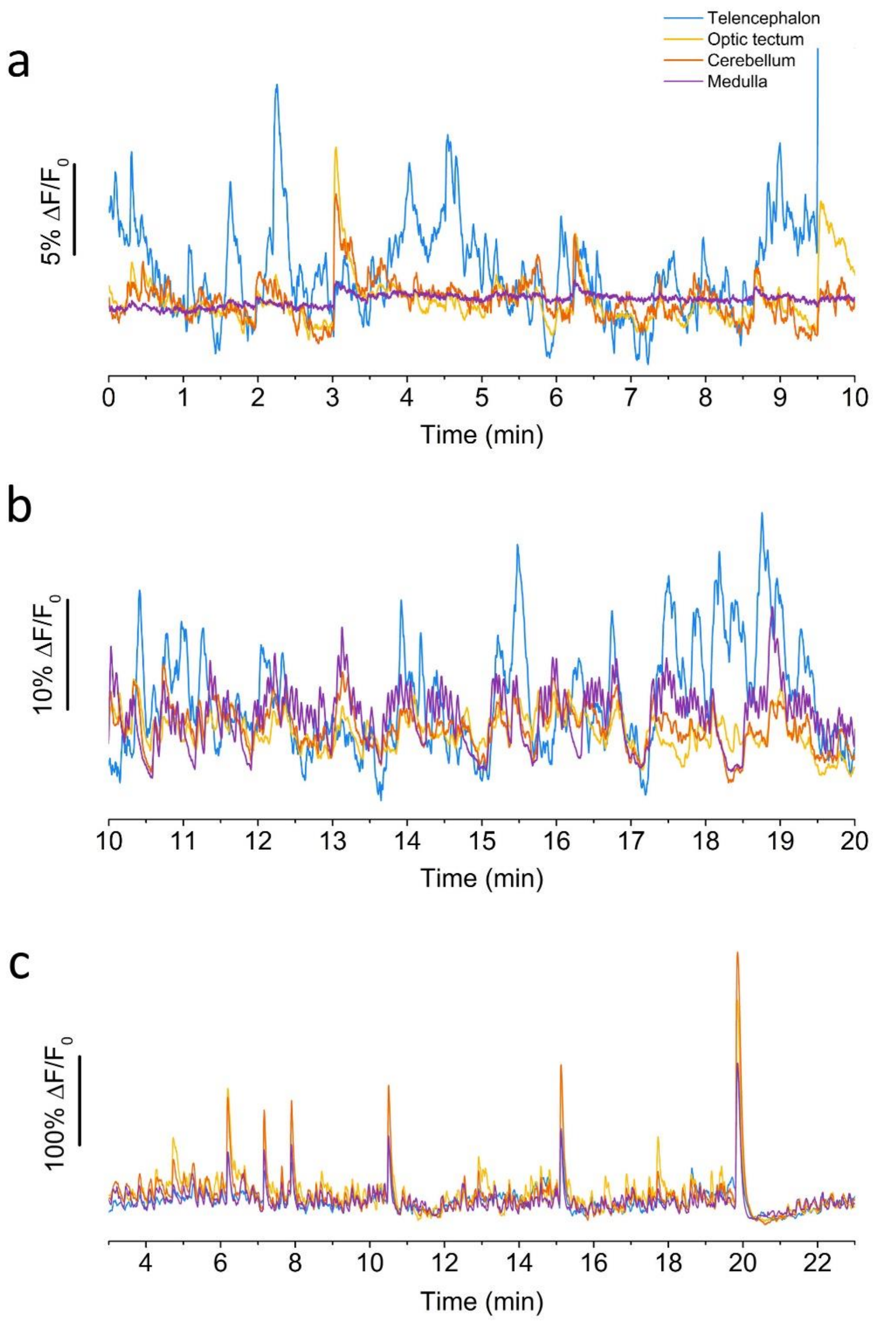

Figure S2. Effect of treatment with paralyzing agent on brain activity. Fluorescence time traces of the four brain regions highlighted in Fig.1a of larvae paralyzed with 2 mM d-tubocurarine exposed to three different conditions 0 (**a**), 1 (**b**) and 15 mM (**c**) PTZ.

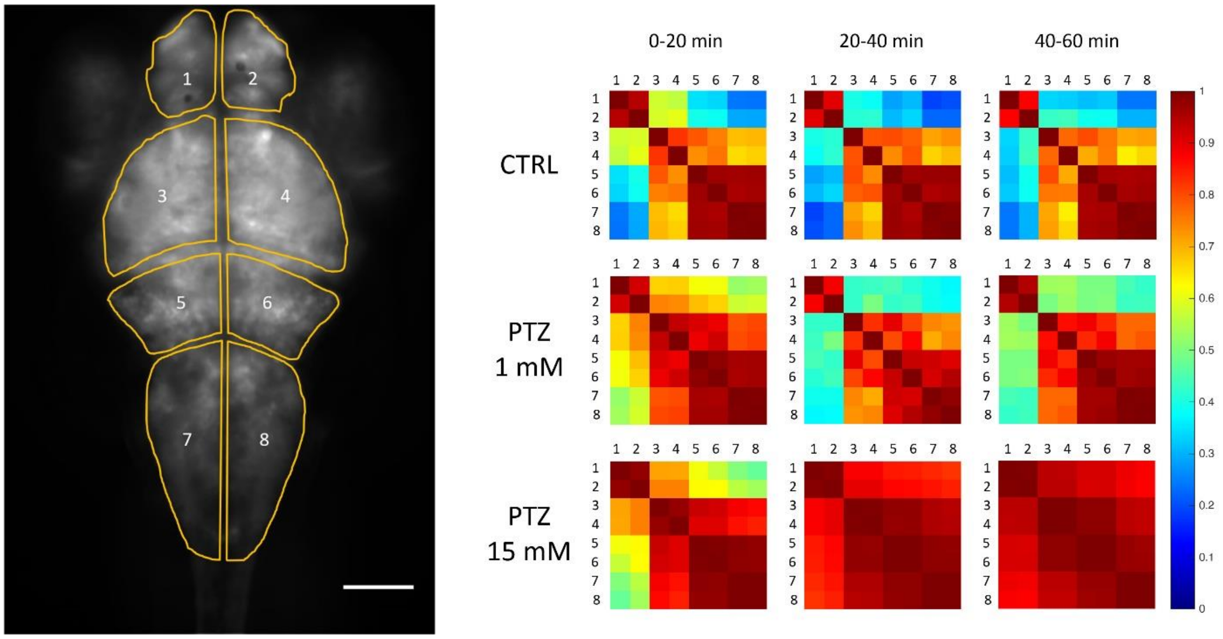

Figure S3. Cross-correlation maps of activity in different brain regions. Left. Regions of interest selected for analysis are shown overlaid with the fluorescence image; scale bar 100 µm. **Right.** Cross-correlation matrices (see Methods) measured at different time intervals during a one-hour recording in different conditions, as indicated. Each matrix shows color-coded mean correlation coefficients of three larvae, exposed to the same condition, during the same timeframe.

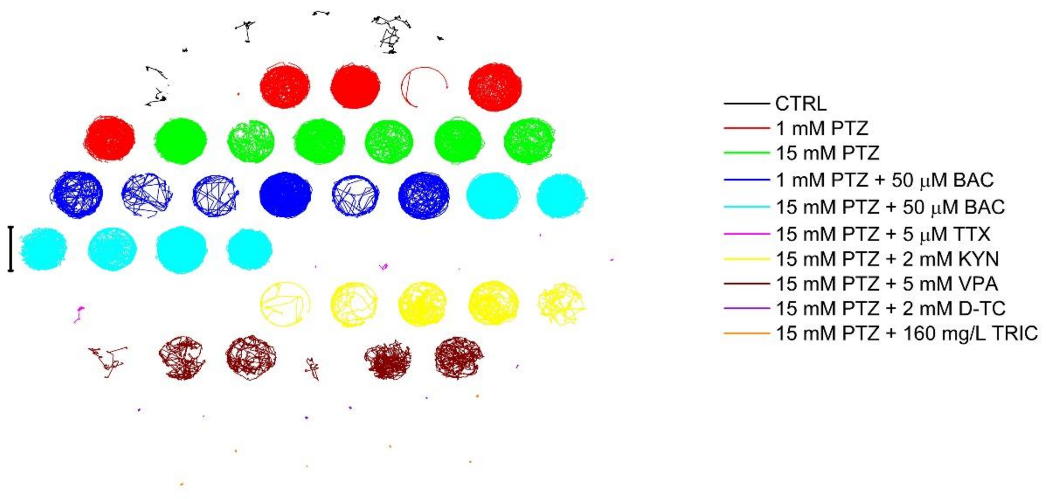

Figure S4. Movement trajectories of larvae in high-throughput assay. For each well the coordinates of the centre of mass of the larva encephalon are plotted for a one-hour measurement. Colours refers to different pharmacological treatments (see legend). Scale bar 7 mm.

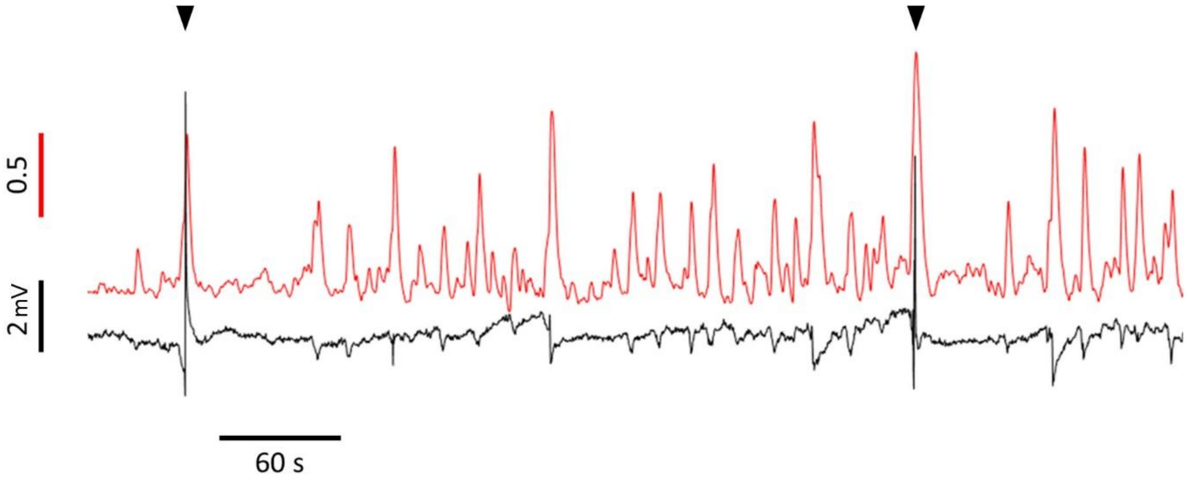

Figure S5. Direct comparison of GCaMP6s fluorescence and electrophysiological recording. The figure shows a representative trace of simultaneous electrographic (black trace) and GCaMP6s fluorescence (red trace) recording on a 4 dpf zebrafish larva with head embedded in 1.5% agarose and free tail (as described in the methods for the samples used in wide field microscopy). The recording was taken 25 minutes after addition of a solution containing 15 mM PTZ in fish water. The extracellular recording trace shows two large ictal bursts (pointed by the arrowheads) separated by inter-ictal activity, as described in the literature (Baraban et al. Neuroscience 131:759, 2005). Comparison of the two traces demonstrates the excellent correspondence between the two measurement methods. Extracellular recordings were performed by inserting a borosilicate electrode, filled with 3 M NaCl, in the optical tectum of the larva. Electrical activity was recorded in current clamp mode with a Multiclamp 700B (Molecular Devices, Sunnyvale, CA). Analog signal was digitized at 10 kHz, low-pass filtered at 1 kHz with a Digidata 1322A acquisition board, commanded with pClamp10 (Molecular Devices). Simultaneous measurement of GCaMP6s fluorescence intensity was achieved with a fluorometric set up consisting of a blue LED excitation source and a photomultiplier tube (Cairn Research). Fluorescence collection area was adjusted manually to contain the entire encephalon of the larva. The photomultiplier signal was acquired and processed as described for electrode signal. Off-line analysis was made with Clampfit 10 (Molecular Devices) and Matlab.

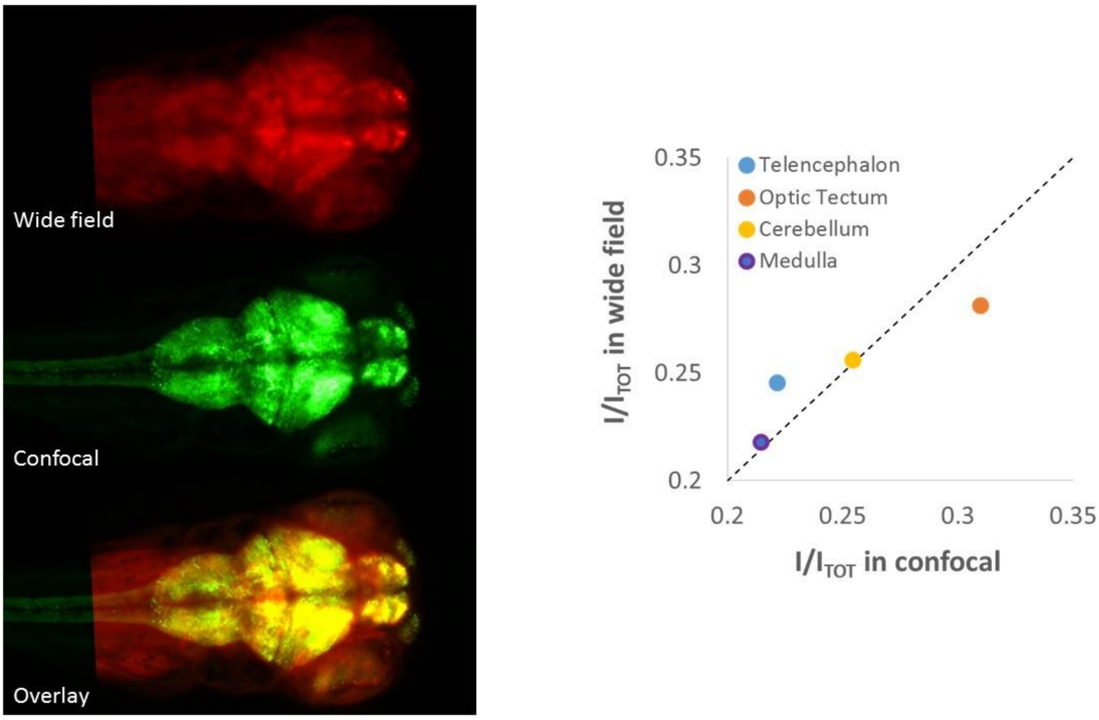

Figure S6. Evaluation of cross-talk between brain regions due to wide-field imaging. Imaging was performed on the same larva in wide field (left, top panel) and confocal (left, middle panel). The confocal image was constructed as the sum of 183 confocal planes (with 2 µm z-steps) encompassing the whole depth of the larva brain. This reconstruction is taken as a good reference for comparison with the wide field image, since each plane does not suffer from contributions of out-of-focus planes from adjacent brain regions at different depths. Comparison of the two images and observation of the overlay (left, bottom panel) demonstrates that the wide field image captures most of the relevant features of the encephalon as imaged with confocal microscope. A quantitative correlation between wide-field versus confocal intensities of the four regions analysed in the paper is shown in the right panel. Intensities are reported as percentage over the total intensity integrated on the whole brain.

Supplementary movie 1. Simultaneous measurements of brain activity and tail movement. The movie shows a 15-minute interval recorded on a larva exposed to 15 mM PTZ. The movie plays at 5x speed with respect to real time. Images are displayed with gamma=0.4 to avoid saturation in the encephalon and still have good contrast on the tail.

Supplementary movie 2. Simultaneous measurements of brain activity and tail movement - control. The movie shows a 2m31s time interval recorded on a larva in fish water (control), exhibiting short and small amplitude tail swings.

Supplementary movie 3. Simultaneous measurements of brain activity and tail movement – 1 mM PTZ. The movie shows a 1m36s time interval recorded on a larva exposed to 1 mM PTZ, exhibiting clustered small amplitude tail swings.

Supplementary movie 4. Simultaneous measurements of brain activity and tail movement – 15 mM PTZ. The movie shows a 30 s time interval recorded on a larva exposed to 15 mM PTZ, exhibiting convulsions characterized by very large brain activity spikes and large tail swings. Images are displayed with gamma=0.4 to avoid saturation in the encephalon and still gave good contrast on the tail.

Supplementary movie 5. High-throughput measurement. The movie shows 13 minutes of recording performed with the high-throughput system. The movie plays at a speed 20x compared to real time. Well content: 1-6 control, 7-12 1mM PTZ, 13-18 15 mM PTZ, 19-24 1 mM PTZ + 50 μM Baclofen, 25-30 15 mM PTZ + 50 μM Baclofen, 31-36 15 mM PTZ + 5 μM Tetrodotoxin, 37 fish water, 38 fish water + 2 mM Kynurenate, 39-43 15 mM PTZ + 2 mM kynurenate, 44-49 15 mM PTZ + 5 mM Valproate, 50-55 15 mM PTZ (after pre-incubation in 2 mM d-tubocurarine for 10 minutes before starting the measurement), 56-61 15 mM PTZ + 160 mg/L Tricaine.

Supplementary movie 6. Head movement during convulsion – larva embedded in agarose. The movie plays at 10x speed compared to real time and demonstrates the movement occurring in the encephalon as a consequence of vibration of the larva in the agarose cylinder during a convulsion.

Supplementary movie 7. Head movement during convulsion – larva with head immobilized in agarose and free tail. The movie plays at 10x speed compared to real time and demonstrates the drastic reduction of movement during convulsion compared to the fully embedded larva shown in Supplementary Movie6. This type of preparation was used in all experiments presented in this work.

Supplementary movie 8. Head movement during convulsion – larva with head immobilized in agarose and free tail, treated with 2 mM curarine. The movie plays at 10x speed compared to real time and demonstrates the full suppression of movement due to the paralyzing agent.
